# Gender differences in cognitive improvements after two months of atypical antipsychotic treatment in first episode schizophrenia

**DOI:** 10.3389/fpsyt.2024.1369532

**Published:** 2024-04-29

**Authors:** Wanyi Li, Xiang Cao, Qian Liang, Yan Li, Chao Zhou, Jinglun Du, Shiping Xie

**Affiliations:** Department of Psychiatry, Affiliated Nanjing Brain Hospital, Nanjing Medical University, Nanjing, Jiangsu, China

**Keywords:** first episode schizophrenia, cognitive function, gender difference, follow-up study, MCCB

## Abstract

**Aims:**

This study aims to explore the gender differences in cognitive improvements after two months of atypical antipsychotic treatment in first episode schizophrenia (FES).

**Methods:**

82 patients with FES, including 50 male patients and 32 female patients, were enrolled in the present study. Positive and Negative Syndrome Scale (PANSS) and MATRICS Consensus Cognitive Battery (MCCB) were respectively conducted to evaluate the clinical symptoms and cognitive function of patients with FES at baseline and after treatment. Repeated measure ANOVA was performed to compare gender differences in cognitive domains scores between baseline and 2-month follow-up. Stepwise liner regression model was performed to explore the effect factors of cognitive improvements in patients.

**Results:**

There was no significant difference in age of onset, education years, PANSS scores, duration of untreated psychosis and Olanzapine equivalent doses between male and female patients (all p > 0.05). In the comparisons of cognition function, male patients exhibited better performance in social cognition compared with female patients at baseline (t = 3.20, *p* < 0.05). After treatment, improvements of attention/vigilance and working memory were both found in male patients and female patients (attention/vigilance, F = 11.867, *p* < 0.05; working memory, F = 18.265, *p* < 0.05). In addition, improvement of speed of information processing was only found in female patients (F = 11.65, *p* < 0.01). Significant interaction between time and gender was found in speed information of processing (F = 4.140, *p* = 0.045). Stepwise liner regression model revealed that improvements of negative symptoms promote improvements of cognitive function in female patients (all *p* < 0.05).

**Conclusions:**

Our findings revealed gender differences of cognitive improvements in patients with FES after 2-month treatment. It provides new evidence for gender differences in cognitive symptoms of schizophrenia, and also provides preliminary clues for further individualized cognitive intervention strategies.

## Introduction

Gender is one of the most important heterogeneity factors in schizophrenia (1). Over the past few decades, numerous studies have explored difference between male and female patients in several aspects of the disease. For example, male patients with schizophrenia appear to exhibit an earlier age of onset, severer negative symptoms and a higher frequency of alcohol/substance abuse than female patients (2, 3). Female patients display superior occupational, interpersonal and psychosocial function compared with male patients (4). Studies also found gender difference in response to antipsychotic drugs. Male patients tend to respond more poorly to antipsychotic medications, exhibit poorer medication adherence, and therefore have more disabilities (5). Inversely, in a 4-18 years follow-up study, female patients with schizophrenia had both better remission of symptoms and functional outcomes after treatments (6). Gender-related characteristics may play a crucial role in the progression and outcome of schizophrenia. Identifying these differences benefits to specify and implement gender-specific intervention strategies.

As the core features of schizophrenia, cognitive deficits occur early and persist across the course of schizophrenia (7, 8), which may be associated with long-term disability and heavy economic burden on society (9). Several studies have devoted to investigate gender difference of cognitive function in schizophrenia. Evidence indicated that gender differences exhibit in the whole course of schizophrenia, including the prodromal, acute and chronic stages (10, 11). Zhao et al (12) have demonstrated that female patients with FES performed better in speed of processing and verbal learning than male patients. However, Zhang et al (13) also reported no gender difference in the comparisons of cognitive function in first episode schizophrenia. Given the inconsistent results on gender difference in cognitive function, one of the reasons may be the heterogeneity of sample size and methods of analyses. The other may be the different tools of cognitive assessment used in the previous studies. Measurement and Treatment of Schizophrenia Cognition Research (MATRICS) Consensus Cognitive Battery (MCCB) (14, 15), which is considered the gold standard for assessing cognitive function in patients with schizophrenia. Based on these reason, this study adopted appropriate assessment tools to further explore gender differences in cognitive function of schizophrenia.

It’s worth noting that previous studies have shown that gender difference in outcome indicators may affect by the time of evaluation. One of the studies reported that more female patients reached a state of recovery compared to male patients after a 5-year follow-up (16). A 10-year follow-up study indicated that gender differences in outcome were attenuated (17, 18). However, the majority of previous studies have mostly employed long-term longitudinal designs (up to 1 year), and the results of short-term study on gender difference in cognitive improvements remain unclear. Study on gender difference in cognitive function after short-term treatment is beneficial to the formulation of individualized intervention strategies for early schizophrenia. Previous studies have reported about 91.4% schizophrenia with FES had improvements in cognitive function after 2 months of atypical antipsychotic treatment (19). And 2 months is seen as a early time window to observe cognitive improvements (20, 21). Up to now, no study has investigated gender difference in cognitive improvements after 2 months of atypical antipsychotic treatment in Chinese Han population. Whether this cognitive improvement may be influenced by gender remains unknown.

In the present study, the main objective was to investigate gender difference in cognitive improvements of schizophrenia with FES after 2-month treatment. We hypothesize that there is gender difference in cognitive improvement of schizophrenia after 2-month treatment and this difference may be related to the improvement of clinical symptoms.

## Materials and methods

### Subjects

Patients with FES were recruited from the inpatient and outpatient of Nanjing brain hospital, Jiangsu, China, from January 2017 to September 2023. Patients were diagnosed by two psychiatrists using the DSM-V who were experienced and involved in the usual care process of patients. All patients meets the following criteria: (1) right handed; (2) age between 16 to 45; (3) Intelligence quotient (IQ)>70 the Wechsler Adult Scale of Intelligence (WAIS) was adopted to test the Intelligence Quotient (IQ); (4) Duration of untreated time ≤ 24 months, never treated with antipsychotic medication or physical therapy. Exclusion criteria included: (1) Mental retardation or other serious mental disorders; (2) Serious physical diseases; (3) History of drug or alcohol abuse.

The study was approved by the Medical Research Ethics Committee of the Affiliated Brain Hospital of Nanjing Medical University(No. 2021-KY075-01). All participants provided written informed consent.

### Nueropsychologic and Clinical measurement

Basic clinical measurements such as age of onset, education years, duration of untreated psychosis were provided by patients and their caregivers. In this study, cognitive assessment was performed using the MCCB, comprised of seven cognitive domains, including speed of information processing, attention and vigilance, verbal learning, working memory, problem solving, visual learning and social cognition (22, 23). Clinical symptoms assessment was performed using the Positive and Negative Syndrome Scale (PANSS), comprising positive symptoms, negative symptoms and general symptoms, and carried out by two experienced psychiatrists (24). All patients were medicated with atypical antipsychotic medication based on routine clinical practice. Seventeen patients were treated with risperidon, thirteen with olanzapine, twenty-one with aripiprazole, fourteen with paliperidone and seventeen with amisulpiride. All drug doses are converted to olanzapine equivalents (25), and cumulative antipsychotic dose was defined as the sum of all daily doses from the start of antipsychotic therapy to the day of retesting MCCB at 2 months follow-up.

### Statistical analyses

Statistical analyses were performed using Statistical Package for the Social Sciences version 25.0 (SPSS 25.0). The normality of data distribution was assessed with the Kolmogorov-Smirnov test and Shapiro-Wilk test. Mann-Whitney U test was adopted to compare difference of education years, age of onset and duration of untreated psychosis. Independent t-test was used to compare PANSS scores, MCCB scores at baseline and olanzapine equivalent doses between male and female patients. Repeated measure ANOVA was performed to compare cognitive domain scores between groups. Stepwise liner regression model was performed to explore the effect factors of cognitive improvements in patients.

## Results

### Demographic and clinical characteristics

A total of 82 schizophrenia with FES (52 males, 30 females) were enrolled in the present study. Compared to normative data from the Chinese population (26), patients with FES showed deficits in all cognitive domains (all *p* < 0.05, see **Table 1** for details).

**Table 1:** Cognitive impairments in schizophrenia with FES.

	N	Mean (SD)	Controls Mean	*t*	*p*
Speed of processing	82	38.45 (11.12)	50	-9.39	0.00
Attention/vigilance	82	39.04 (11.01)	50	-9.01	0.00
Working memory	82	36.48 (10.69)	50	-11.45	0.00
Verbal learning	82	37.56 (11.27)	50	-9.98	0.00
Visual learning	82	41.85 (12.28)	50	-6.00	0.00
Problems solving	82	46.76 (11.42)	50	-2.57	0.01
Social cognition	82	33.46 (10.83)	50	-13.81	0.00
Composite scores	82	32.37 (12.39)	50	-12.88	0.00

FES, First episode schizophrenia.

N, Total number of schizophrenia.

Mean:,All data were stated as Mean (SD).

t, Independent t-test.

P, p < 0.05 indicates statistical significance.

There were no significant differences in age of onset, education years, PANSS scores (including positive scores, negative scores and general psychopathology scores) and duration of untreated psychosis between male and female at baseline. There was also no significant differences in Olanzapine equivalent doses between groups (all *p* > 0.05). In the comparisons of cognition function, male patients exhibited better performance in social cognition compared with female patients at baseline (t = 3.20, *p* < 0.05, see **Table 2** for detalis).

**Table 2:** Gender differences in demographic and clinical characteristics in schizophrenia with FES.

	Male (n=50)	Female (n=32)	*t/U*	*P*
Age of onset (years)^a^	22.50 (18.75-30.25)	21.00 (18.00-29.00)	-0.78	0.43
Education years ^a^	13.00 (11.00-15.25)	13.00 (11.00-15.00)	-0.46	0.64
Dup (months)^a^	6.00 (3.75-12.00)	10.00 (6.00-12.00)	-0.75	0.45
Olanzapine equivalent doses ^b^	14.08 (4.41)	12.45 (3.87)	1.70	0.09
Baseline symptoms severity				
PANSS-positive scores ^b^	21.72 (3.36)	21.31 (3.28)	0.53	0.59
PANSS-negative scores ^b^	18.98 (4.05)	19.31 (4.66)	-0.14	0.88
PANSS-general scores ^b^	44.10 (3.85)	43.97 (3.65)	0.15	0.87
PANSS-total scores ^b^	84.78 (7.35)	84.38 (9.30)	0.21	0.82
Baseline cognitive impairment severity				
Speed of information processing ^b^	39.14 (11.26)	37.38 (10.99)	0.70	0.48
Attention/vigilance ^b^	37.88 (11.81)	40.84 (9.52)	-1.19	0.23
Verbal learning ^b^	37.74 (11.43)	37.28 (11.20)	0.18	0.85
Working memory ^b^	36.60 (10.78)	36.28 (10.72)	0.13	0.89
Visual learning ^b^	40.94 (11.97)	43.28 (12.83)	-0.84	0.40
Problem solving ^b^	45.70 (11.04)	48.41 (11.98)	-1.04	0.29
Social cognition ^b^	36.22 (11.10)	29.16 (8.97)	3.02	0.00*

FES, first episode schizophrenia.

Dup, duration of untreated psychosis.

PANSS, Positive and Negative Syndrome Scale.

^a^Independent t-test; ^b^Mann-Whitney U test.

### Gender difference in cognitive function at baseline and 2-month follow-up

**Figure 1:**
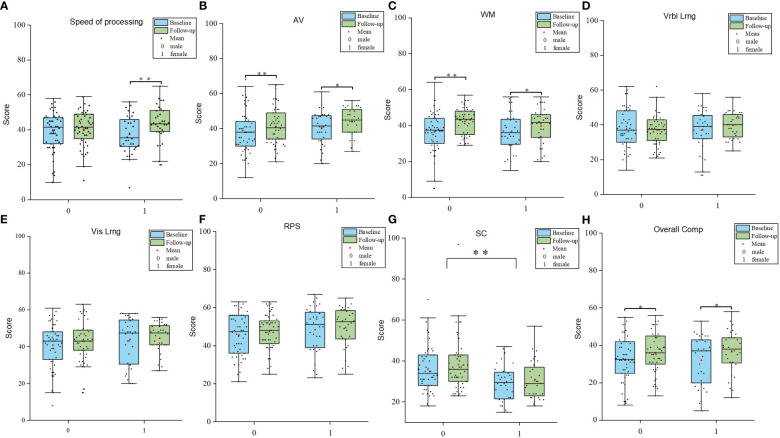
Gender differences in cognitive improvements. **(A-H)** Comparisions of different cognitive domains between male and female patients. AV Attention/Vigilance; WM Working memory; Vrbl lrng Verbal learning; Vis lrng Visual learning; PRS Problem solving; SC Social cognition. *p < 0.05, **p < 0.01.

After 2-month treatment, both male and female patients exhibited improvements in attention/vigilance and working memory (attention/vigilance, F = 11.867, *p* < 0.05; working memory, F = 18.265, *p* < 0.05). Furthermore, only female patients showed improvement in speed of information processing (F = 11.65, *p* < 0.01). All enrolled patients demonstrated significant enhancements in overall cognitive function after 2 months of treatment with atypical antipsychotics (F = 9.708, *p* < 0.01). Notably, a significant interaction between time and gender was observed in terms of speed of information processing (F = 4.140, *p* = 0.045). (see **Figure 1** for details).

### Gender difference in effect factors of cognitive improvements

Stepwise liner regression model revealed that improvements of negative symptoms promote improvements of cognitive function in female patients. (all *p* < 0.05, see details in **Table 3**). No significant effect factor of cognitive improvements was found in male patients.

**Table 3:** Gender difference in effect factors of cognitive improvements in schizophrenia.

Cognitive improvement	PANSS symptoms improvement	B	*β*	*t*	*p*
Speed of information processing	Negative symptoms	-1.90	-0.713	-5.56	<.01
Attention/vigilance	Negative symptoms	-1.26	-0.59	-4.09	<.01
Verbal learning	Negative symptoms	-2.10	-0.68	-5.08	<.01
Working memory	Negative symptoms	-1.27	-0.54	-3.60	<.01
Visual learning	Negative symptoms	-2.10	-0.68	-5.08	<.01
Problem solving	Negative symptoms	-1.57	-0.58	-3.90	<.01
Social cognition	Negative symptoms	-1.47	-0.58	-3.95	<.01
Overall Composite scores	Negative symptoms	-2.56	-0.77	-6.75	<.01

PANSS, Positive and Negative Syndrome Scale.

## Discussion

In the present study, we explored gender differences in cognitive improvements of first episode schizophrenia after 2 months of treatment. The main findings emerged 1) Male patients had better social cognition than female patients at baseline. 2) Improvement of speed of processing was only found in female patients. Significant interaction between time and gender was found in speed information of processing. 3) Negative symptoms improvement may promote cognitive improvements in female patients.

Evidence shows that individuals with schizophrenia exhibit different psychiatric symptoms based on gender. Males tend to experience more severe negative symptoms, while females tend to experience more severe positive symptoms (13, 27–29). However, the present study found no gender differences in psychiatric symptoms of first episode schizophrenia, which was inconsistent with previous research findings. A global study had revealed that gender differences in psychotic symptoms displayed regional heterogeneity, possibly associate with psychosocial and cultural factors in different residential regions (30). Additionally, the heterogeneity in sample size and assessments tools adopted may contribute to potential discrepancies in research outcomes.

Previous studies have reported cognitive impairments in patients with schizophrenia during the early stages of this illness (7, 31–33). In our study, we found that first episode schizophrenia exhibited extensive cognitive deficits compared to normative data from the Chinese population. These findings provide additional evidence to support this viewpoint and further enhance its reliability. When analyzing the data by gender grouping, a noteworthy trend emerged among patients, showing that males scored higher in social cognition, indicating better social function compared to females. However, the conclusions regarding gender differences in social cognition still remain controversial. For instance, previous researches indicated that males exhibited superior social cognition compared to females at the early and chronic stages of schizophrenia (27, 34). It is important to note that some studies suggested there was no significant difference in social cognitive function between gender (13). A potential explanation for the inconsistent of our results could be the complexity of the social cognition functional construct (35). Prior investigations have revealed that social cognition encompasses diverse abilities, including theory of Mind, emotional processing (EP), social perception, and attribution style (36). However, the MCCB used in the present study primarily assessed the EP aspect of individual social cognition. Furthermore, a 10-year follow-up study provided evidence that male patients with schizophrenia exhibited poorer social cognition than female patients (37).Combined with the results of this study, it is possible to speculate that the female advantage in social cognition might become apparent later in the disease course. However, relevant studies need to be further verified.

The present study found that only female patients exhibited improvements in processing speed, which consistent with findings from previous research (34, 38, 39). Notably, a rodent study simulating schizophrenia-like behavior also revealed that female mice demonstrated better processing speed than male mice (40). Nevertheless, the pathophysiological mechanism underlying gender differences in speed of processing improvements in schizophrenia remains inconclusive. Currently, the most widely accepted explanation is that estrogen has a protective impact on cognitive function in patients with schizophrenia (41–43). And previous study also reported that there is an inverse correlation between psychotic symptoms and estrogen levels (44). Some females with psychotic tendencies experience more pronounced symptoms after menopause (45, 46). Estrogen diminished psychotic symptoms by modulating major neurotransmitter systems associated with schizophrenia, such as the dopamine signaling system. However, this protection primarily prevents further cognitive deterioration over time in schizophrenia, rather than preventing damage from the disease itself, as evidenced by the absence of gender differences in processing speed improvements among patients with FES at baseline. Furthermore, the superior response of female patients to antipsychotic drugs may provide an additional explanation. Specifically, estrogen influences the activity of CYP1A2, a major enzyme involved in olanzapine metabolism (47). Consequently, With the same dosage, the drug plasma concentration of olanzapine in female patients with schizophrenia was higher than that in males with schizophrenia and produce better results (48). Numerous prior studies have demonstrated that gender may change brain structure through mediating neurodevelopment, resulting in various psychiatric symptom (49–52). Future magnetic resonance findings should be included to explore gender differences in cognitive structure of processing speed in schizophrenia.

Previous studies have identified a convergence between negative symptoms and cognitive impairments in individuals with schizophrenia (53, 54). Oliver et al. (55) investigated that cognitive changes were associated with negative factors in a one-year follow-up study. Chen et al. also reported that negative symptoms were associated with longitudinal changes of cognitive function (56). Overlapping etiologies may exited between negative symptom subdomains and cognitive function. Cognitive function and clinical symptoms are not independent but affects each other. However, we only found that negative symptoms improvements were correlated with cognitive improvements in female patients, which suggested there may be gender specificity in curative effect. The earlier the intervention for negative symptoms, the greater may be cognitive improvements in female patients.

Cognitive remediation (CR) is an important therapeutic method for cognitive impairments in schizophrenia. CR as a type of behavioral training aims to improve cognitive function with the goal of persistence and generalization in everyday life. CR is effective in promoting the improvement of cognitive function, social and daily living function in patients with schizophrenia (57). Exsting MRI research conducted on individuals in the early stages of schizophrenia has revealed that CR interventions are associated with structural and functional alterations in the brain, particularly in the frontal and limbic regions (58, 59). CR may have a slowing or reversing effect on progressive brain volume deterioration in the early stages of schizophrenia, especially in areas critical to higher cognitive processes (58, 60). Vita et al. also reported that long-term improvement of psychosocial functioning after CR can be observed with significant gender differences, and females exihibited more significant improvements in cognitive function (61). Currently, only a few studies have concentrated on gender differences in cognitive improvements after CR intervention, and the results have been inconsistent. Thus, in the future studies, gender differences in cognitive improvement and brain structure changes after CR interventions need to be further explored at the early stage of the disease by using multimodal techniques. Longitudinal studies are also needed to clarify gender differences in cognitive training and whether its positive effects persist over time.

### Limitation

This research has some limitations. First, our study lasted only 2 months, which is much shorter than previous studies. The findings from longitudinal investigations suggested that the duration of 1-3 years may not be sufficient to detect significant changes in cognitive function (62). In our future studies, we intend to conduct ongoing follow-up assessments of the participants who have been enrolled in our study. Second, our research is an observational study, the pathological mechanisms of gender differences in cognitive function in schizophrenia are not yet clear. We need to incorporate magnetic resonance data for further study. Third, the study employed inconsistent drug interventions. Previous research has demonstrated varying degrees of improvement in cognitive function depending on the type of antipsychotic medication utilized (63). Fourth, the sample size after the gender-stratified analysis was relatively small, and our stratified results should be validated in larger studies in the future. Finally, women’s neuropsychological performance has been shown to fluctuate with the menstrual cycle, which was not controlled for in our study (64).

## Conclusion

In summary, our results indicated that gender differences of cognitive function exhibited at baseline and 2-month follow-up, which provides some clues for the personalized treatment of schizophrenia patients. Understanding these differences may help develop more precise treatment strategies for individuals with schizophrenia.

## Data availability statement

The original contributions presented in the study are included in the article/**Supplementary Material**. Further inquiries can be directed to the corresponding authors.

## Ethics statement

The studies involving humans were approved by the Medical Research Ethics Committee of the Affiliated Brain Hospital of Nanjing Medical University. The studies were conducted in accordance with the local legislation and institutional requirements. The participants provided their written informed consent to participate in this study.

## Author contributions

WL: Writing – review & editing, Writing – original draft, Methodology, Data curation. XC: Writing – original draft. QL: Writing – review & editing, Data curation. YL: Writing – review & editing, Data curation. CZ: Writing – review & editing, Methodology, Formal Analysis. JD: Writing – review & editing, Supervision, Resources, Funding acquisition. SX: Writing – review & editing, Supervision, Resources, Funding acquisition.
