# Supplementary material for: Gender differences in cognitive improvements after two months of atypical antipsychotic treatment in first episode schizophrenia
Source: Front Psychiatry. 2024 Apr 29;15:1369532. doi: 10.3389/fpsyt.2024.1369532 (PMC11089100; doi:10.3389/fpsyt.2024.1369532)
Supplement: Supplementary file 1 [file Table_1.docx]

**Supplementary materials**

**Table S1: Gender differences in cognitive improvements**

|  | Male(n=50) | | | Female(n=32) | | | Gender F (P value) | | Ge×TP F(P value) |
| --- | --- | --- | --- | --- | --- | --- | --- | --- | --- |
|  | Baseline | 2-month | *p* | Baseline | 2-month | *p* | Baseline F(p value) | 2-month F(p value) |  |
| Speed of processing | 39.14(11.26) | 40.70(9.86) | 0.31 | 37.38(10.99) | 43.97(9.96) | 0.00* | 0.48 | 0.14 | 0.04* |
| Attention/vigilance | 37.88(11.81) | 41.28(10.89) | 0.00** | 40.84(9.52) | 44.00(7.74) | 0.03* | 0.23 | 0.22 | 0.89 |
| Verbal learning | 37.74(11.43) | 37.10(8.83) | 0.69 | 37.28(11.20) | 39.87(7.34) | 0.19 | 0.85 | 0.14 | 0.21 |
| Working memory | 36.60(10.78) | 42.34(7.39) | 0.00** | 38.28(10.72) | 39.84(10.07) | 0.03* | 0.89 | 0.20 | 0.32 |
| Visual learning | 40.94(11.96) | 42.96(10.42) | 0.23 | 43.28(12.83) | 44.66(8.55) | 0.51 | 0.40 | 0.44 | 0.81 |
| Problem solving | 45.70(11.04) | 47.34(9.11) | 0.26 | 48.41(11.98) | 49.53(10.73) | 0.53 | 0.29 | 0.35 | 0.82 |
| Social cognition | 36.22(11.10) | 38.18(13.32) | 0.21 | 29.16(8.97) | 30.56(9.30) | 0.47 | 0.00** | 0.00** | 0.82 |
| Composite scores | 32.50(12.90) | 32.16(13.14) | 0.03* | 36.12(13.14) | 36.69(11.26) | 0.02* | 0.90 | 0.81 | 0.72 |

All present p value. Significant results are shown in Table S 1.

Ge, Gender; TP, follow-up time

* p＜0.05

** p＜0.01
